# 

**Published:** 1882-10

**Authors:** 


THE article by J. L. Wortman is taken from the advance sheets of the new edition of W. H. Clarke's work on horses' teeth.

### BOOKS AND PAMPHLETS RECEIVED.

The Antiseptic Treatment of Wounds. By W. T. Briggs, M.D. Reprint from *Nashville Journal of Medicine and Surgery*.

The Treatment of Subacute and Chronic Gout. By Alexander Hadden, M.D. New York City: 8vo, pp. 20.

Studies from the Biological Laboratory of Johns Hopkins University. Baltimore: Vol. II, No. 3.

ANNALES ET BULLETIN DE LA SOCIÉTE DE MEDICINE DE GAND.

JAHRESBERICAT DER KONIGLICHEN THIERARZNEISCHULE ZU HANNOVER. Herausgegeben von dem Lehrer—Collegium redigirt von dem Director Dr. Dammann. Vierzehnter Bericht, 1880–82.

MONTREAL VETERINARY COLLEGE. Sixteenth Annual Announcement, Session 1882–83.

KANSAS CITY REVIEW OF SCIENCE AND INDUSTRY. Edited by Theodore S. Case. Monthly.

JOHNS HOPKINS UNIVERSITY CIRCULARS. No. 17. August, 1882.

THE VETERINARY JOURNAL. London.

DER ZOOLOGISCHE GARTEN. Frankfurt.

WOCHENSCHRIFT FUR THIERHEILKUNDE UND VIEHZUCHT. Augsburg.

SCHWEIZERISCHES ARCHIV FUR THIERHEILKUNDE UND THIERZUCHT. Bern.

REPERTORIUM DER THIERHEILKUNDE. Stuttgart.

ARCHIV FUR WISSENSCHAFTLICHE UND PRACTISCHE THIERHEILKUNDE. Berlin.

LA PRESSE MEDICALE. Paris.

THE CHICAGO MEDICAL JOURNAL AND EXAMINER. Chicago.

THE MEDICAL RECORD. New York.

THE COLLEGE AND CLINICAL RECORD. Philadelphia.

ANNALS OF ANATOMY AND SURGERY. Brooklyn.

NEW ENGLAND MEDICAL MONTHLY. Newton, Conn.

CHICAGO MEDICAL REVIEW. Chicago.

ST. LOUIS CLINICAL RECORD. St. Louis.

VIRGINIA MEDICAL MONTHLY. Richmond.

NASHVILLE JOURNAL OF MEDICINE AND SURGERY. Nashville.

THE BLACKSMITH AND WHEELWRIGHT. New York.

NATIONAL LIVE STOCK JOURNAL. Chicago.

THE BREEDER'S GAZETTE. Chicago.

THE CULTIVATOR AND COUNTRY GENTLEMAN. Albany.

INDIANA FARMER. Indianapolis.

MIRROR AND FARMER. Manchester, N. H

TRUTH. San Francisco.

THE WEEKLY DROVER'S JOURNAL. Chicago

